# Competitive and Recreational Running Kinematics Examined Using Principal Components Analysis

**DOI:** 10.3390/healthcare9101321

**Published:** 2021-10-03

**Authors:** Wenjing Quan, Huiyu Zhou, Datao Xu, Shudong Li, Julien S. Baker, Yaodong Gu

**Affiliations:** 1 Faculty of Sports Science, Ningbo University, Ningbo 315211, China; nbuquanwenjing@gmail.com (W.Q.); zhouzhouhuiyu@163.com (H.Z.); xudatao3@gmail.com (D.X.); 2 Faculty of Engineering, University of Pannonia, H-8201 Veszprém, Hungary; 3 Savaria Institute of Technology, Eötvös Loránd University, 9700 Szombathely, Hungary; 4 School of Health and Life Sciences, University of the West of Scotland, Glasgow G72 0LH, UK; 5 Department of Sport and Physical Education, Hong Kong Baptist University, Hong Kong 999077, China; jsbaker@hkbu.edu.hk

**Keywords:** principal component analysis, kinematics data, long-distance running, joint angle

## Abstract

Kinematics data are primary biomechanical parameters. A principal component analysis (PCA) of waveforms is a statistical approach used to explore patterns of variability in biomechanical curve datasets. Differences in experienced and recreational runners’ kinematic variables are still unclear. The purpose of the present study was to compare any differences in kinematics parameters for competitive runners and recreational runners using principal component analysis in the sagittal plane, frontal plane and transverse plane. Forty male runners were divided into two groups: twenty competitive runners and twenty recreational runners. A Vicon Motion System (Vicon Metrics Ltd., Oxford, UK) captured three-dimensional kinematics data during running at 3.3 m/s. The principal component analysis was used to determine the dominating variation in this model. Then, the principal component scores retained the first three principal components and were analyzed using independent *t*-tests. The recreational runners were found to have a smaller dorsiflexion angle, initial dorsiflexion contact angle, ankle inversion, knee adduction, range motion in the frontal knee plane and hip frontal plane. The running kinematics data were influenced by running experience. The findings from the study provide a better understanding of the kinematics variables for competitive and recreational runners. Thus, these findings might have implications for reducing running injury and improving running performance.

## 1. Introduction

Long-distance running is a convenient sport that is popular with numerous runners globally [1,2]. With the increased number of runners, over-use running injuries have increased. According to epidemiological investigations, the running injuries risk has increased to as high as 79% yearly [3,4]. It also appears that recreational runners have a higher risk of lower limb running injuries than competitive runners [5]. Most of the running studies for different runners have analyzed direct static parameters, such as kinematics [6,7,8], running fatigue [9], plantar pressure [10] and the kinetics of gait running [11]. However, the running mechanisms involved in competitive running and recreational running are still unclear. As a result, biomechanical research should consider investigating any differences in running experience and effects on performance.

A number of studies have investigated biomechanical running experience. It was noted that running experience might influence the risk of running injuries. It has been proposed that novice runners were more prone to lower limb injuries than recreational runners per 1000 h running [12]. Following fatigue running, the results indicated that the forward trunk lean and hip abduction significantly increased during the running stance phase in recreational compared to competitive runners [9]. Peter R. et al. demonstrated that elite runners had more significant knee flexion during the swing stance, and regular runners showed greater plantar flexion and dorsiflexion during the swing stance. However, only sagittal plane kinematics parameters were studied. All the runners were highly trained and included good runners and experienced marathon runners with mean marathon times of 2:34:40 [13]. The hip joint is a complex structure, and greater hip flexion and hip ROM for novice runners has been observed. However, the ankle and knee did not differ between novice and experienced runners’ kinematics data [11].

Some researchers have also proposed that running experience was not able to influence running biomechanics variables. The previous study demonstrated that running kinematics were influenced by age, which might cause knee flexion reduction and increase contact time during running. With increased years of running experience, the kinematics variables of spatiotemporal variables, kinematics variables and lower limb joint ankle were unchanged [14]. In addition, Schmitz et al. compared the kinematics and kinetics variables between novice runners and experienced runners. The study found no significant differences between the two groups of runners, including impact peak, loading rate, and hip kinematics [6].

In recent years, new analysis techniques, such as machine learning, have been used to analyze running biomechanics. A support vector machine and principal component scores have been applied to the kinematics parameters to determine the differences between competitive and recreational runners. The results favored the support vector machine (SVM) to achieve cross-fold classification accuracies of between 80.00–100% in knee flexion angle in the sagittal plane, ankle angle in the frontal plane and pelvis in the sagittal plane [15]. Eneida et al. compared the three-dimensional kinematics of the foot-ankle and ground reaction forces (GRF) by a SVM for runners of different experience. It was been found that, with increased running experience, the dorsiflexion of the first metatarsophalangeal joint and plantarflexion angles were significantly decreased [16]. Similarly, previous studies have found that PCA techniques may distinguish between frontal and sagittal kinematics data for healthy and pathological groups’ gait patterns [17].

Principal component analysis is a method used to identify data patterns and express data to highlight the similarities and differences between data. It has been widely used in many research fields involving data analysis and preprocessing [18]. Principal component analysis was demonstrated as a multivariate statistical analysis for human movement data and gait analysis [19,20,21]. The model of principal component analysis is to reduce the dimensionality of the original data into a small number of unrelated composite indicators to replace the original indicators. Through the compression and dimensionality reduction of the original data, the time required for data analysis can be reduced, and the original complex problems can be simplified. The dimensionality reduction can make the data retain as much information of the original data as possible to reflect the real situation and maximize information. Each principal component has its corresponding eigenvalue, and the size of the eigenvalue reflects the variance contribution of the principal component. The larger the eigenvalue, the stronger the resolution of the original data, i.e., the more information the principal component contains about the original data [22].

The PCA algorithm has been proposed for biomechanics applications. Many previous studies have shown that principal component analysis could distinguish different gait dynamics more sensitively than traditional parameter analysis techniques [23,24,25,26]. For example, Benno M. Nigg et al. used a principal component analysis and a support vector machine to demonstrate gender differences in sagittal and frontal kinematic PCA during the running swing and stance phases [16]. Additionally, Phinyomark et al. demonstrated that young runners influenced the knee and ankle sagittal angle during the initial running contact and swing phase using principal component analysis [27]. Deluzio et al. used this technique to identify the difference in biomechanics parameters for knee osteoarthritis populations [19] and normal populations [24]. Robbins et al. compared the joint angle for high- and low-caliber hockey players using principal component analysis [28]. However, little research has classified the differences between competitive and recreational runners’ kinematics variables using principal component analysis.

Few studies have found kinematics variable differences between different level runners in previous research. However, in some investigations, PCA can extract the key features for the joint angle, joint moment and joint force and distinguish the groups differently. A principal component analysis is a reduction technique that might reduce the time-wave dimensionality. To compare the variables of running lower limb kinematics, the purpose of the present study was to utilize PCA to identify the ankle, knee and hip joint differences during the running phase between competitive runners and recreational runners. We used the PCA waveform analysis to extract primary features that can quantify differences for competitive runners and recreational runners. We hypothesized that lower limb joint angle differences would exist between experienced runners and reactional runners during the running stance phase.

## 2. Materials and Methods

### 2.1. Participants

Twenty healthy male competitive runners (height: 171.6 ± 3.5 cm; age: 26.7 ± 2.1 years; body: 65.2 ± 4.8 kg; running experience: 6.5 ± 1.3 years) and twenty male recreational runners (height: 163.2 ± 2.7 cm; age: 25.4 ± 1.9 years; body: 51.8 ± 3.6 kg; running experience: 1.4 ± 0.7 years) were recruited from the running club and the university running association to participate in this study. Competitive runners had to satisfy the following conditions: rearfoot strike pattern, run at least 15 km per week and have running experience of more than five years [15]. All the competitive runners experienced marathon competition. Recreational runners had to satisfy the following conditions: rearfoot strike pattern, run 2 to 5 km per week [11]. They did not have the training experience required for long-distance running. Prior to the test, all participants were without any lower limb extremity injuries and any medical history of musculoskeletal injuries in the last year. A written informed consent form was obtained from each participant before the study explaining the purpose of the study and experimental procedures. The Ethics Committee approved (Approval Number: RAGH20210221) the study of Ningbo University.

### 2.2. Test Protocol

Prior to running data collection, all participants were required to wear uniform running shoes (Anta, Flashedge, China) and tight pants and t-shirts, and were fully familiarized with testing procedures. Thirty-six retroreflective markers were fixed to anatomical landmarks. Then, the spherical reflective (14 mm) markers were put on the bilateral lower limbs. The marker position defined by skeletal landmarks included: right/left anterior superior iliac spine, right/left posterior superior iliac spine, right/left femur lateral epicondyle, right/left femur medial epicondyle, right/left first and fifth metatarsal heads, right/left distal interphalangeal joint of the second toe, right/left medial and lateral malleoli, right/left medial and lateral epicondyle of the femur. Clusters of 4 markers were placed laterally on the right and left thigh and shank segments [29].

The participants were required to stand on the force plate following the reflective marker placement for static model data collection. In addition, before the experiment, all the participants were asked to run and jog for warm-up activities on the ground. Participants were then asked to right foot run through the force plate at a speed of 3.3 m/s. Kinematics data were captured using an eight-camera motion system (Vicon Metrics Ltd., Oxford, UK) at a sampling rate of 200 Hz. Each participant completed five successful running trials over a 15 m runway at 3.3 m/s in a standard biomechanics laboratory. A speed measuring instrument (smart speed, Fusion Sport Inc., Burbank, CA, USA) was placed on both sides of force plate to control running speed. A successful run required the participants to run with the entire right foot placed on the force plate at the running speed of 3.3 m/s.

### 2.3. Data Process and Analysis

The 6DOF lower limb model was used to calculate three-dimensional kinematic variables [30]. The original data were identified, track repaired and named using the integrated system supporting software Vicon Nexus 1.8.6, then the file was finally exported “*.c3d” into the Visual3D. Kinematic data were processed and analyzed using Visual 3D (v6; C-Motion, Inc., Germantown, MD, USA). The reflective markers were low-pass filtered at a cut-off frequency of 14 Hz by a fourth-order Butterworth filter [31]. Initial contact and toe-off of the ground were defined as the running stance period. The initial contact and toe-off were determined when the vertical GRF crossed 30 N threshold level [9]. Joint angle (ankle, hip, knee) was calculated using Cardan angles in the sagittal, frontal and transverse planes [32].

### 2.4. Principal Component Analysis Methods

PCA can use data compression and dimensionality reduction methods to present the original data as a set of orthogonal variables called principal components (PCs) in a new hyperplane and then classify the prominent waveform characteristics [24]. Therefore, we used this technique to extract the primary characters of variation in the lower limb kinematics between the competitive runners and recreational runners in this study.

PCA was used to identify dominant modes of variation for the three angles (hip, knee, ankle) in three-dimensional planes (sagittal, frontal, transverse), calculating waveforms in nine analyses (three for ankle, three for knee and three for hip). The reason for using this approach is that this method was more sensitive in terms of separating the difference in the lower limb angles change in three-dimensional planes. At first, the captured motion kinematics data for the sagittal, frontal and transverse planes were normalized to 101 time points of running stance (Version: R2019a, The MathWorks, Natick, MA, USA).

For each PCA analysis, data were created into a n×p matrix (*X*) 101×200 (n×p) matrix (*X*).

(1) $$X_{n\times m}=\left[\begin{array}{cccc} x_{11} & x_{12} & \cdots & x_{1p}\\ x_{21} & x_{22} & \cdots & x_{2p}\\ \ldots & \ldots & \cdots & \ldots \\ x_{n1} & x_{n2} & \cdots & x_{np} \end{array}\right]=\left(X_{1},X_{2},\cdots ,X_{p}\right)$$

where *p* represents 200 trials (40 subjects × 5 trials), and where *n* represents 101 data points over running stance phase.

First, data preprocessing was performed on the original data *X* to remove the influence of features with larger overall values on the proportion of variance in the calculation. The mean of each joint angle was calculated $\vec{X_{mean}}\;$ when participants ran through the force plate during the running stance phase.

(2) $$\vec{X_{mean}}\;=\frac{1}{p}\sum_{i=1}^{p}x_{j}^{\left(i\right)}$$

Then, $x_{j}^{\left(i\right)}$ was replaced with $x_{j}^{\prime \left(i\right)}$. This makes the mean of each feature 0.

(3) $$x_{j}^{\prime \left(i\right)}=x_{j}^{\left(i\right)}-\vec{X_{mean}}$$

After the original data matrix *X* was preprocessed, it was expressed as:

(4) $$X^{\prime }=\left(\begin{array}{cccccc} x_{1}^{\left(1\right)} & x_{2}^{\left(1\right)} & \cdots & x_{j}^{\left(1\right)} & \cdots & x_{n}^{\prime \left(1\right)}\\ x_{1}^{\prime \left(2\right)} & x_{2}^{\prime \left(2\right)} & \cdots & x_{j}^{\left(2\right)} & \cdots & x_{n}^{\left(2\right)}\\ \vdots & \vdots & \ddots & \vdots & \ddots & \vdots \\ x_{1}^{\left(i\right)} & x_{2}^{\left(i\right)} & \cdots & x_{j}^{\left(i\right)} & \cdots & x_{n}^{\left(i\right)}\\ \vdots & \vdots & \ddots & \vdots & \ddots & \vdots \\ x_{1}^{\left(p\right)} & x_{2}^{\left(p\right)} & \cdots & x_{j}^{\left(p\right)} & \cdots & x_{n}^{\prime \left(p\right)} \end{array}\right)$$

The covariance between each dimension of ‘*X* was calculated, and the obtained n×n  matrix formed the covariance matrix of *X*′:

(5) $$\Sigma =\frac{1}{m-1}{X\prime }^{T}X^{\prime }$$

The eigenvalues and eigenvectors of the covariance matrix were then calculated. First, the eigenvalue of and corresponding eigenvectors Σ in the fifth equation were calculated. The eigenvectors were then arranged in the order of the eigenvalues to obtain the corresponding eigenvector matrix.

(6) $$U=\left(u^{\left(1\right)},u^{\left(2\right)},\ldots ,u^{\left(n\right)}\right)$$

To reduce the n-dimensional raw data to d-dimensions, the first d columns of the principal component matrix calculated in Equation (6) were extracted to obtain the dimensionality reduction matrix:

(7) $$U_{\mathrm{reduce}\;}=\left(u^{\left(1\right)},u^{\left(2\right)},\ldots ,u^{\left(d\right)}\right)$$

The original data matrix *X* was multiplied with the reduced matrix $\;U_{\mathrm{reduce}\;}$ in Equation (7) to obtain the final reduced data matrix *Z*:

(8) $$Z=XU_{\mathrm{reduce}\;}$$

### 2.5. Principal Components Numbers

The eigenvalues of the covariance matrix and its corresponding eigenvectors reflect a linear transformation process of the original data. The eigenvectors represent the main transformation direction, while the eigenvalues represent the degree of transformation of the corresponding eigenvectors. It can also be described that the eigenvectors represent the angles of the matrix space features, and the corresponding eigenvalues represent the variance of each angle. However, there are several common criteria for selecting principal components: cumulative contribution margin criterion and Bartlett test criterion Kaiser method. In this paper, the number of principal components was selected using the cumulative contribution rate. The number of principal components can be selected according to the actual needs, for example, if the cumulative contribution of the first k principal components reaches 85%. Then, the number of principal components is determined ask Cumulative Contribution Ratio (CCR) is obtained from the following equation. In the present study, the first three PCs were conducted because they represent the primary variables of kinematics data. We used the PCA approach by MATLAB (Version: R2019a, The MathWorks, Natick, MA, USA) [33].

(9) $$\Sigma_{i=1}^{k}\;\lambda_{i}/\Sigma_{i=1}^{n}\;\lambda_{i}$$

where *k* is the number of selected principal components, *n* is the total number of all principal components.

### 2.6. Statistical Analysis

Principal component scores of waveforms were calculated using the mean and standard deviation for sagittal ankle plane, frontal ankle plane, ankle transverse, knee sagittal, knee frontal plane, knee transverse, hip sagittal, hip frontal and hip transverse. PC1 represented the overall amplitude and shape in three planes, and PC2 was the range of motion for the ankle, knee and hip joint. PC3 represented the initial contact angle during the running stance.

To compare the differences between the competitive runners and recreational runners, we used independent *t*-tests using SPSS23.0 software. We compared principal component scores for three planes waveforms. Statistically significant *p*-values were set at 0.005.

## 3. Results

In the nine kinematics waveforms, principal components models included the nine joint angles waveforms in Table 1. The three principal components (PCs) were chosen in each model. We compared the differences in principal components scores for the competitive runners and recreational runners by independent *t*-tests; the *p*-values and data are described in Table 2.

**Figure 1:**
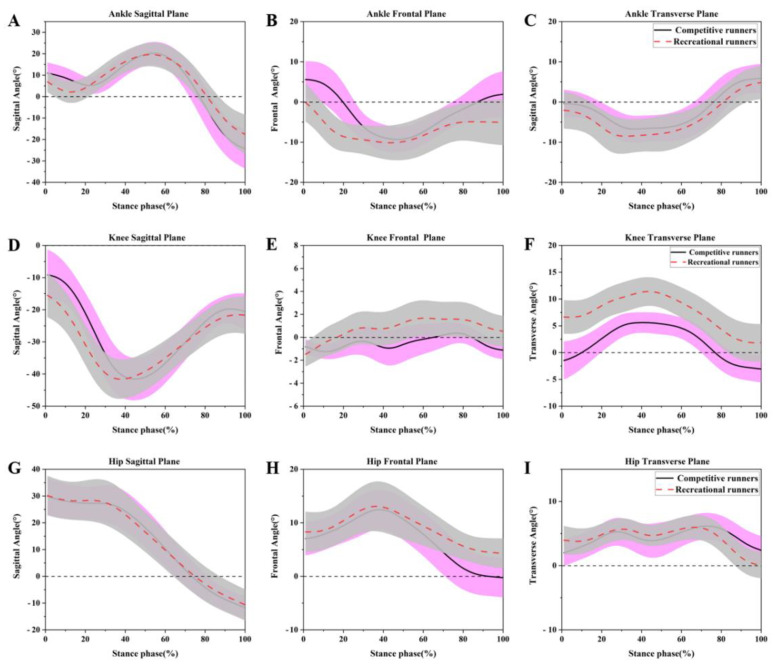
The means and standard deviations for ankle angles in (**A**) sagittal plane, (**B**) frontal plane, (**C**) transverse plane, knee angles in the (**D**) sagittal plane, (**E**) frontal plane, (**F**) transverse plane, and hip angles in the (**G**) sagittal plane, (**H**) frontal plane, (**I**) transverse plane during the running stance phase for the competitive runners (black, solid lines) and recreational runners (red, dashed lines).

All the joint ankle means and original waveforms for the sagittal plane, frontal plane and transverse plane are depicted in Figure 1.

### 3.1. Ankle Results

**Figure 2:**
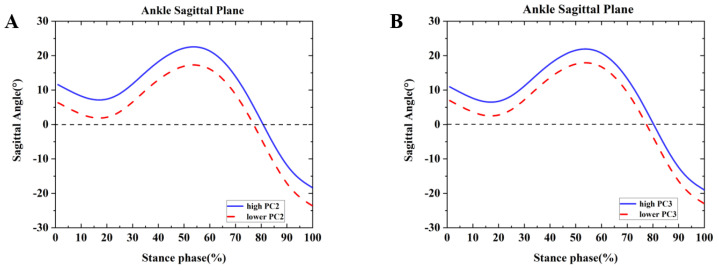
Principal components are retained for the ankle. (**A**) The waveform differences between competitive runners and recreational runners of reconstruction principal using the high and lower PC2-scores in the ankle sagittal plane. (**B**) The waveform differences between competitive runners and recreational runners of reconstruction principal using the high and lower PC3-scores in the ankle sagittal plane.

Differences were found between the competitive and recreational runners for the ankle sagittal plane (Table 2 and Figure 2). For the PC2 in the sagittal plane, all the positive values were equal values (Figure 2A,B). In the sagittal plane, the two principals depicted the overall magnitude of ankle dorsiflexion during the running stance phase. The high waveform indicated that the ankle had more dorsiflexion than the lower waveform. The recreational runners showed negative principal PC2 in Figure 2A and Table 2 (*p* = 0.001). Therefore, the recreational runners exhibited a smaller dorsiflexion angle than the competitive runners during the running stance. In addition, high PC3 indicated a more significant change in the dorsiflexion during the initial contact (0–80%) to plantarflexion during the swing phase in Figure 2B and Table 2 (*p* = 0.001). The recreational runners exhibited greater dorsiflexion during the initial contact than the competitive runners.

**Figure 3:**
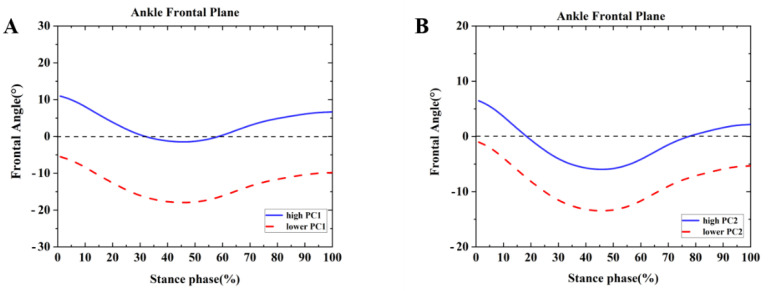
Principal components are retained for the ankle. (**A**) The waveform differences between competitive runners and recreational runners of reconstruction principal using the high and lower PC1-scores in the ankle frontal plane. (**B**) The waveform differences between competitive runners and recreational runners of reconstruction principal using the high and lower PC2-scores in the ankle frontal plane.

Differences were found between the competitive and recreational runners for the ankle frontal plane (Table 2 and Figure 3). For the PC1 in the frontal plane, the high waveform showed a larger inversion than the lower. The recreational runners showed negative principal PC1 in Figure 3A and Table 2 (*p* = 0.001). Therefore, the recreational runners exhibited a smaller inversion angle than the competitive runners during the running stance. In addition, high PC2 indicated range of motion changes during the running stance phase. The competitive runners showed negative PC2 in Figure 3B and Table 2 (*p* = 0.001). The competitive runners exhibited a smaller ROM than the recreational runners during the running stance in the ankle frontal plane.

**Figure 4:**
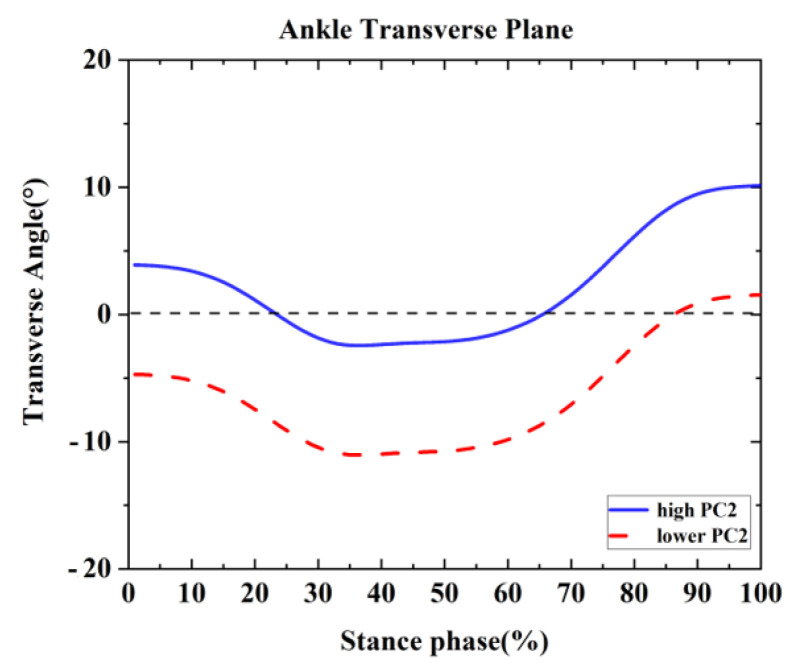
Principal components are retained for the ankle. The waveform differences between competitive runners and recreational runners of reconstruction principal using the high and lower PC2-scores in the ankle transverse plane.

Differences were found between the transverse ankle plane’s competitive and recreational runners (Table 2 and Figure 4). For the PC2 in the transverse plane, the high waveform showed a larger range of motion than the lower waveform. The competitive runners showed a negative PC2 in Figure 4 and Table 2 (*p* = 0.001). The competitive runners exhibited a smaller ROM than the recreational runners during the running stance in the transverse ankle plane.

### 3.2. Knee Results

**Figure 5:**
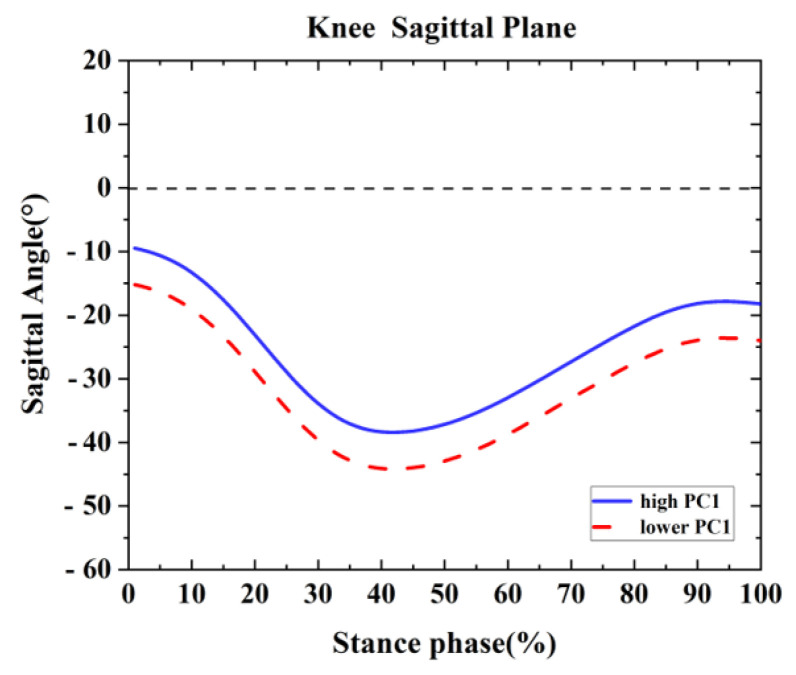
Principal components are retained for the knee. The waveform differences between competitive runners and recreational runners of reconstruction principal using the high and lower PC1-scores in the ankle sagittal plane.

One difference was found between the competitive and recreational runners for the knee sagittal plane (Table 2 and Figure 5). For the PC1 in the knee sagittal plane, the high waveform showed larger flexion than the lower. The recreational runners showed larger principal PC1 in Figure 3A and Table 2 (*p* = 0.001). Therefore, the recreational runners exhibited a larger knee flexion angle than the competitive runners during the running stance.

**Figure 6:**
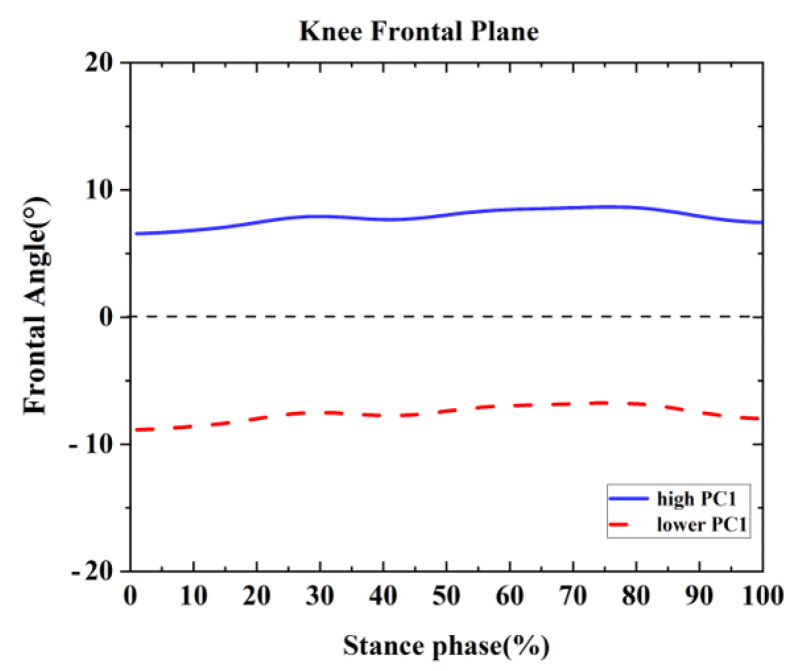
Principal components are retained for the knee. The waveform differences between competitive runners and recreational runners of reconstruction principal using the high and lower PC1-scores in the frontal plane.

One difference was found between the competitive and recreational runners for the sagittal knee plane (Table 2 and Figure 5). For the PC1 in the frontal knee plane, the high waveform showed larger adduction than the lower. The recreational runners showed smaller principal PC1 in Figure 6 and Table 2 (*p* = 0.001). Therefore, the recreational runners exhibited less knee adduction angle than the competitive runners during the running stance.

**Figure 7:**
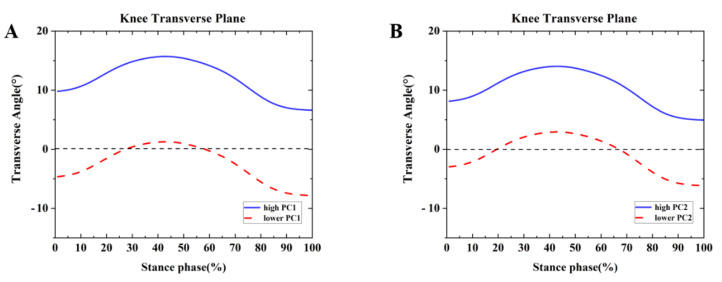
Principal components are retained for the knee. (**A**) The waveform differences between competitive runners and recreational runners of reconstruction principal using the high and lower PC1-scores in the transverse plane. (**B**) The waveform differences between competitive runners and recreational runners of reconstruction principal using the high and lower PC2-scores in the transverse plane.

Differences were found between the competitive and recreational runners for the ankle frontal plane (Table 2 and Figure 7. For the PC1 in the transverse plane, the high waveform showed larger internal rotation than the lower. The recreational runners showed positive principal PC1 in Figure 7A and Table 2 (*p* = 0.001). Therefore, the recreational runners exhibited a greater internal rotation angle than the competitive runners during the running stance. In addition, high PC2 indicated range of motion changes during the running stance phase. The competitive runners showed negative PC2 in Figure 7B and Table 2 (*p* = 0.001). The competitive runners exhibited greater ROM than the recreational runners during the running stance in the knee transverse plane.

### 3.3. Hip Results

**Figure 8:**
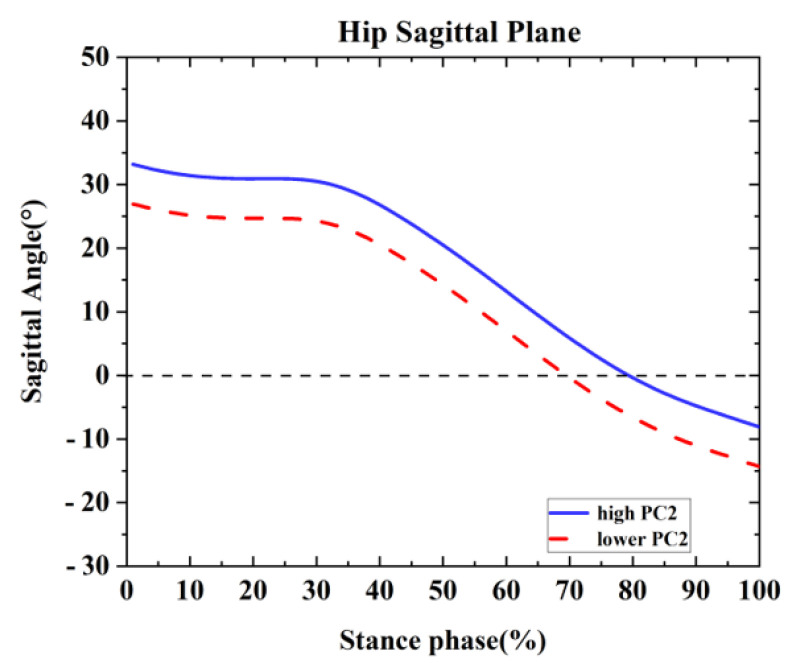
Principal components are retained for the hip. The waveform differences between competitive runners and recreational runners of reconstruction principal using the high and lower PC2-scores in the sagittal plane.

One difference was found between the competitive and recreational runners for the hip sagittal plane (Table 2 and Figure 8). For the PC2 in the hip sagittal plane, the high waveform showed larger flexion than the lower. The recreational runners showed positive principal PC1 in Figure 8 and Table 2 (*p* = 0.001). Therefore, the recreational runners exhibited a larger hip flexion angle than the competitive runners during the running stance.

**Figure 9:**
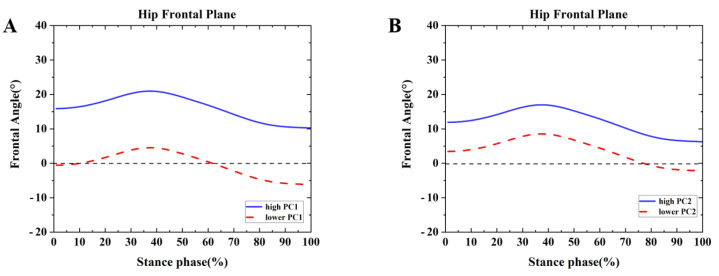
Principal components are retained for the hip. (**A**) The waveform differences between competitive runners and recreational runners of reconstruction principal using the high and lower PC1-scores in the frontal plane. (**B**) The waveform differences between competitive runners and recreational runners of reconstruction principal using the high and lower PC2-scores in the frontal plane.

Differences were found between the competitive and recreational runners for the hip frontal plane (Table 2 and Figure 9). For the PC1 in the frontal plane, the high waveform showed larger adduction than the lower. The recreational runners showed positive principal PC1 in Figure 9A and Table 2 (*p* = 0.001). Therefore, the recreational runners exhibited a greater adduction angle than the competitive runners during the running stance. In addition, high PC2 indicated the range of motion changes during the running stance phase. The recreational runners showed negative PC2 in Figure 9B and Table 2 (*p* = 0.001). Thus, the competitive runners exhibited greater ROM than the recreational runners during the running stance in the hip frontal plane.

**Figure 10:**
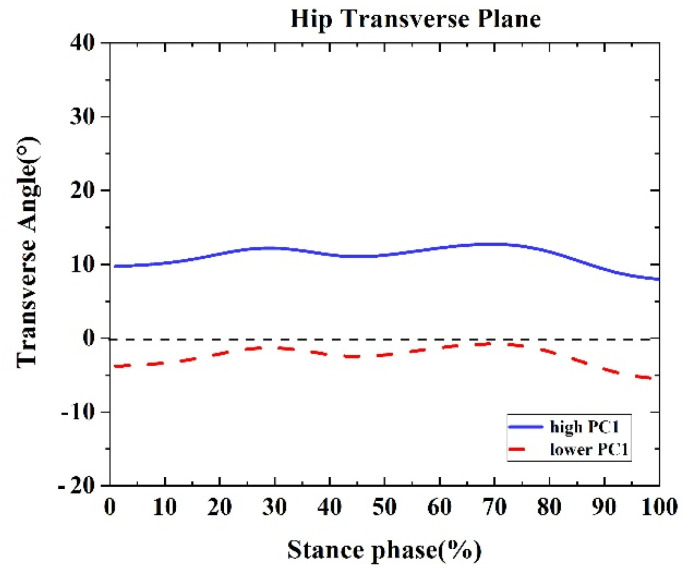
Principal components are retained for the hip. The waveform differences between competitive runners and recreational runners of reconstruction principal using the high and lower PC1-scores in the transverse plane.

One difference was found between the competitive and recreational runners for the hip transverse plane (Table 2 and Figure 10). For PC1 in the hip transverse plane, the high waveform showed a larger internal rotation than the lower. The recreational runners showed negative principal PC1 in Figure 10 and Table 2 (*p* = 0.001). Therefore, the recreational runners exhibited less hip internal rotation angle than the competitive runners during the running stance.

## 4. Discussion

The present study aimed to compare the variables of running lower limb kinematics using the principal component analysis approach between competitive runners and recreational runners. Our hypothesis was that lower limb joint angle differences would exist between competitive runners and recreational runners during the running stance phase. This study has observed that lower limb kinematics data derived from PCA might be more sensitive than traditional technology. This resulted in differences in lower limb joint kinematics variables during the running between the competitive and recreational runners being revealed.

The basic idea of principal component analysis is to reduce the dimensionality of the original data into a small number of unrelated composite indicators to replace the original indicators. Through the compression and dimensionality reduction of the original data, the time required for data analysis can be reduced, and the original complex problems can be simplified. The dimensionality reduction can make the data retain as much information of the original data as possible to reflect the real situation to the maximum. Each principal component has its corresponding eigenvalue, and the size of the eigenvalue reflects the variance contribution of the principal component. The larger the eigenvalue, the stronger the degree of analysis of the original data, that is, the more information the principal component contains about the original data. This method allows the analysis of the motion patterns over time and is well suited for biomechanical analysis [34]. In addition, PCA-based variability measures appear to be more sensitive to differences in study conditions than peak amplitude-based variability measures [26].

Previous studies have found greater plantarflexion and dorsiflexion of good runners during the running swing stance phase. This study was consistent with previous research that compares the elite runners and recreational runners’ sagittal kinematics variables, which were reflected in ankle sagittal plane PC2 [13]. However, in previous peak data analysis, there was no significant difference in the ankle kinematics [14]. The recreational runner has a smaller initial contact angle in dorsiflexion during the running swing phase; however, a larger inversion ankle joint appears for competitive runners. In addition, the competitive runners have a smaller ROM in the ankle frontal and transverse plane. This suggests that recreational runners are more prone to running injury risks. As for the sagittal knee plane, the recreational runners have demonstrated a larger knee flexion angle, which is observed in the knee sagittal plane PC1. It has been reported that increased knee flexion during running might reduce the risk of knee injury [35]. The greater knee flexion angle was found in the competitive runners, which suggests that greater knee flexion in the competitive runners appears to be a protective adaptation. In addition, the recreational runners during the running stance phase increased knee internal rotation, which was reflected in the knee transverse PC1. Increased femoral internal rotation of the knee suggests an increased risk of knee injury, and the tibial syndrome is a high-incidence injury in recreational runners [36].

The hip joint performs a crucial role in lower limb motion, and instability of the hip joint is considered an essential mechanism of lower limb injury [37]. Increased internal and external rotation and internal abduction of the hip joints are associated with patellofemoral pain and iliotibial tract syndrome [38,39]. Our results for the hip sagittal plane PC2 were consistent with previous studies using traditional analysis techniques that reported greater hip flexion and hip ROM for novice runners [11]. Peak adduction and hip ROM in the frontal plane reflect in the hip frontal plane PC1. During the running stance phase, competitive runners have the strength of hip muscle force, improving hip stability during the running phase. This result confirmed that the waveform’s mean hip transverse magnitude was greater than that of the competitive runners for the hip transverse PC scores. The findings elucidated the main features for competitive and recreational runners, particularly in the hip, ankle and knee joints. These results may have practical implications for the prevention-related injuries of competitive runners and recreational runners.

While acknowledging the results of this study, some limitations should be considered. Firstly, kinetic parameters were not collected, and competitive runners and recreational runners may have a different ground reaction force, although all the runners were rearfoot strikers. Secondly, we used the same running speed to capture the kinematics data without considering the influence of different running speeds on running kinematics in lower limb variables. Lastly, in this study, all the runners were male. In future research, focus and attention should consider different genders to compare the kinematics and kinetics variables of the lower limb joints.

## 5. Conclusions

The present study aimed to utilize PCA to identify the kinematics parameters differences during the running stance phase between competitive runners and recreational runners. Using the principal component scores, the critical features for the joint angle were obtained, which demonstrated differences in the lower limb kinematics for the competitive and recreational runners. The recreational runners’ essential differences included a smaller dorsiflexion angle, initial dorsiflexion contact angle, ankle inversion, knee adduction, range motion in the frontal knee plane and hip frontal plane during the running stance phase. The findings from the study provide a better understanding of the kinematics variables for competitive and recreational runners. Thus, these findings might have implications for reducing running injury and improving running performance.

## Figures and Tables

**Table 1:** The first three principal components for the sagittal, frontal and transverse angle waveforms during the running stance phase.

Variables	PC1 (%)	PC2 (%)	PC3 (%)	Total (%)
Ankle sagittal plane	85.1	6.9	4.0	96
Ankle frontal plane	67.9	14.1	6.9	88.9
Ankle transverse plane	71.8	18.5	2.7	93
Knee sagittal plane	80.9	8.3	3.7	92.6
Knee frontal plane	59.5	14.1	9.1	82.7
Knee transverse plane	52.1	30.8	6.2	89.1
Hip sagittal plane	88.46	9.72	1.14	99.32
Hip frontal plane	67.6	17.9	6.6	92.1
Hip transverse plane	45.6	20.0	10.5	76.1

**Table 2:** The first three principal components (PCs) are retained according to the 90% trace criterion for the competitive runners and recreational runners; *p*-value 0.005 represents significance.

Gait Measure	PC	Competitive Runners	Recreational Runners	*p*-Value	Effect Size
Ankle Sagittal	PC1	0.57(10.04)	0.57(8.34)	0.379	0.001
	PC2	2.41(1.03)	−2.41(1.07)	0.001 *	0.917
	PC3	−0.72(1.92)	0.72(1.82)	0.001 *	0.359
Ankle Frontal	PC1	4.15(8.93)	−4.15(4.69)	0.001 *	0.503
	PC2	−2.87(2.94)	2.87(1.75)	0.001 *	0.765
	PC3	0.20(2.14)	−0.20(3.05)	0.275	0.076
Ankle Transverse	PC1	1.34(8.26)	−1.34(8.51)	0.024	0.158
	PC2	−4.17(1.12)	4.17(0.93)	0.001 *	0.970
	PC3	−0.15(1.75)	0.15(1.50)	0.195	0.092
Knee Sagittal	PC1	1.57(9.84)	0.06(7.80)	0.013	0.085
	PC2	−2.70(1.27)	−0.01(0.57)	0.001 *	0.807
	PC3	0.05(1.82)	0.01(2.05)	0.719	0.010
Knee Frontal	PC1	7.53(0.57)	0.04(2.16)	0.001 *	0.921
	PC2	0.28(4.76)	0.02(2.35)	0.285	0.034
	PC3	0.24(2.09)	0.01(3.73)	0.259	0.038
Knee transverse	PC1	−4.58(5.65)	4.60(5.52)	0.001 *	0.635
	PC2	3.95(1.67)	−3.94(5.26)	0.001 *	0.710
	PC3	−0.44(3.37)	0.45(0.86)	0.012	0.178
Hip sagittal	PC1	−0.15(9.57)	0.15(9.28)	0.824	0.016
	PC2	−3.02(0.79)	3.02(0.66)	0.001 *	0.972
	PC3	0.20(1.03)	−0.20(1.07)	0.009	0.187
Hip frontal	PC1	−2.39(9.23)	2.39(6.25)	0.001 *	0.290
	PC2	3.85(2.43)	−3.85(0.33)	0.001 *	0.911
	PC3	0.22(2.27)	−0.22(2.85)	0.216	0.127
Hip transverse	PC1	6.50(2.34)	−6.50(0.98)	0.001 *	0.964
	PC2	0.02(5.29)	−0.02(3.49)	0.944	0.004
	PC3	−0.61(4.18)	0.61(1.71)	0.008	0.188

Note: * significant waveform reconstruction differences between competitive runners and recreational runners (*p* ≤ 0.005).

## Data Availability

The data that support the findings of this study are available on reasonable request from the corresponding author.
